# A study of the perceived risks, benefits and barriers to the use of SDD in adult critical care units (The SuDDICU study)

**DOI:** 10.1186/1745-6215-11-117

**Published:** 2010-12-03

**Authors:** BH Cuthbertson, J Francis, MK Campbell, L MacIntyre, I Seppelt, J Grimshaw

**Affiliations:** 1 Department of Critical Care Medicine, Sunnybrook Health Sciences Centre, 2075 Bayview Av, Toronto, Ontario, Canada; 2 Health Services Research Unit, Health Sciences Building, University of Aberdeen, Foresterhill, Aberdeen, UK; 3 Clinical Epidemiology, Ottawa Health Research Institute (on behalf of the Canadian Critical care Trials Group), Ottawa, Canada; 4 Nepean Hospital, Penrith, New South Wales, Australia on behalf of the Australia and New Zealand Intensive Care Society Clinical trials Group and the George Institute for International Health, Sydney, Australia; 5 Clinical Epidemiology, Ottawa Health Research Institute, Ottawa, Canada

## Abstract

**Background-:**

Hospital acquired infections are a major cause of morbidity and mortality and markedly increased health care costs. Critically ill patients who require management in an Intensive Care Unit are particularly susceptible to these infections which are associated with a very high mortality. Selective decontamination of the digestive tract (SDD) may reduce these infections and improve mortality but it has not been widely adopted into practice. We aim to 1. Clarify reasons why clinicians have avoided implementing SDD into clinical practice despite the current best-evidence 2. Describe barriers to SDD implementation and 3. Identify what further evidence is required before full scale clinical implementation would be considered appropriate and feasible.

**Methods-:**

We have developed an international 'multi-lens' approach to investigate SDD from several perspectives. In case studies we will identify accounts of implementation of SDD in practice, in terms of the behaviours performed by the full range of individual clinicians, accounts of how SDD was first introduced into the Unit and specific content that may be used to populate the content of behaviour change techniques to be used in an implementation intervention and procedures to consider in order to deliver an implementation trial. In a 4 round Delphi study we will identify the range of stakeholders' beliefs, views and perceived barriers relating to the use of SDD. We will generate hypotheses about key beliefs about SDD and will inform the feasibility of any future randomised controlled trial. In large-scale nationwide postal questionnaire surveys of the state of current practice we will identify the factors predicting acceptability of an effectiveness or implementation trial using, and informed by, the theoretical domains structure. In semi-structured interviews with active international clinical trialists we will assess the feasibility of a randomised controlled trial and identify challenges and barriers to undertaking research in the field of SDD research.

**Discussion:**

We believe these methods will allow us to determine whether clinical implementation trials or further large effectiveness trials are required before full scale implementation into clinical practice.

## Background

Each year in the UK 140,000 patients are admitted to an intensive care unit (ICU) and of these almost 60,000 will die within a year of admission. Hospital acquired infections (HAI) are a major clinical problem for modern health services as they are associated with morbidity and mortality as well as high additional health care costs. Critically ill patients are extremely susceptible to HAI and these are associated with a high additional mortality, prolonged hospital stays and a large health care resource utilisation. Between 20 and 50% of ICU patients suffer from such infections and major efforts are being undertaken to improve these outcomes [1,2].

An intervention that has gained much interest in reducing HAI is selective decontamination of the digestive tract (SDD). SDD involves the application of topical non-absorbable antibiotics to the oropharynx and stomach and a short course of intravenous antibiotics [3-14]. The evidence base relating to SDD is reasonably strong with 12 meta-analyses of 28 randomised controlled studies in the literature [3-14]. Ten of these studies demonstrate a benefit in terms of reducing pneumonia rates and six studies show a specific mortality benefit in all ICU patients or in major subgroups [3]. A very recent large cluster randomised study from the Netherlands demonstrated a 3.5% reduction in adjusted mortality associated with SDD [14]. Problems with these meta-analyses include clinical heterogeneity, lack of blinding and lack of data on compliance with intervention. The Cochrane review of SDD demonstrated an association with reduction in pneumonia, OR 0.32 (0.26-0.38) and death OR 0.75 (0.65-0.87)[3]. None of the published meta-analyses included the recent cluster randomised study [14]. This mortality benefit was present in the more recent randomised studies and is of the magnitude of 3-6% absolute risk reduction (ARR) with a number needed to treat (NNT) of approximately 17 to save one life [3,14]. If this mortality benefit could be realised in UK practice then it could save as many as 2-3000 lives per annum. However, these findings require to be confirmed in other health care environments.

Despite this evidence base, ICU practitioners have not widely adopted this intervention with only 10-15 ICUs (out of 240) reporting that they undertake SDD in the UK [14,15]. Existing surveys of practice have been unable to fully elucidate the reasons for this lack of adoption and implementation [14,15]. Reasons may include fears over encouraging antibiotic resistance, a lack of biological plausibility for the findings and a lack of external validity of the evidence base since most of the existing studies come from countries where infections due to multi-resistant organisms are uncommon [14]. Finally, it is possible that this intervention is so counterintuitive that clinicians will not change their practice regardless of the evidence base or that one clinician group could impede another group, who are in favour of the intervention, from implementing it.

It seems that until there is high quality evidence demonstrating clinical effectiveness, cost-effectiveness and the ecological impact of SDD in a range of health care systems, this intervention is unlikely to be implemented and thus patients will be denied a potentially life saving therapy. However, despite the clear importance of HAI in critically ill patients, and despite a multi-national appreciation and prioritisation of the importance and urgency of this research topic, it remains unclear why this intervention has not been implemented. Little is known about clinicians' beliefs about the existing evidence base, the perceived benefits and risks of SDD in clinical practice, the factors that influence current practice and likely barriers to implementation. Further, it is also unclear whether there is a requirement for further high level evidence of effectiveness before implementation would become acceptable and what sort of study would be feasible and acceptable to clinicians and trialists. This multi-method study will address these issues in three regions (the UK, Canada and Australia/New Zealand).

## Aims

The overall aim of this study is to identify the perceived risks, benefits and barriers to the use of SDD in critical care units. To achieve this aim, the following objectives are proposed (with formal research questions (RQs) listed):

1. To identify and precisely describe the clinical intervention in units and hospitals that deliver SDD:

• **RQ1**: What are the components of the intervention of SDD?

• **RQ2**: How has SDD been implemented and delivered into practice?

2. To identify the range of beliefs, interpretation and views about the current evidence base relating to the use of SDD in key stakeholder groups:

• **RQ3**: What are the views of key stakeholders about the likely positive and negative consequences of implementing SDD in ICUs?

• **RQ4**: What are the views of key stakeholders about the likely *barriers *to implementing SDD in ICUs?

• **RQ5**: What are the views of key stakeholders of the internal/external validity and adequacy of the existing evidence base for SDD?

3. To identify current practice and assess the acceptability of further randomised controlled trials in the field of SDD in a wide group of intensive care clinicians and infectious disease clinicians/medical microbiologists:

• **RQ6**: What are the current practices and intentions of intensivists and infectious disease clinicians/medical microbiologists about SDD?

• **RQ7**: If there are uncertainties in the evidence base, do these clinicians believe they could be addressed in a clinical trial and what research questions, trial design(s) and interventions would be optimal and what predicts these beliefs?

4. To assess the feasibility of a proposed effectiveness randomised controlled trial comparing SDD against a control group in ICUs among international intensive care clinical trialists:

• **RQ8**: What are the likely challenges in undertaking a large multi-national randomised controlled study of SDD in ICU?

## Methods

**Figure 1:**
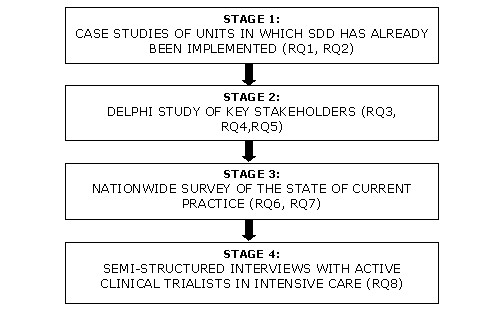
Design of study showing links to research questions

The study will use a 'multi-lens' approach to investigate SDD from several perspectives and in three national regions (the UK, Canada and Australia/New Zealand) to allow greater understanding and international comparisons of the issues that relate to the implementation of this therapy. The investigation will involve four inter-related stages (Figure 1 shows a schema of this study).

### Stage 1

#### Case studies will be conducted in units in which SDD has been implemented (targeting RQs 1 and 2)

This stage will be conducted only in the UK region as there are no adult critical care units in the other regions who use SDD. The case studies will focus on a behavioural analysis of the processes of implementation, based on a modification of Michie and Johnston's [16] advice for specifying clinical behaviour (who, what, to whom, when, how). It will identify the "A,B,C" (antecedents, behaviour, consequences)[17] of each action that is taken in the sequence of behaviours between identifying a patient who may be eligible for SDD, prescribing, supplying, storing, communicating, administering (to the patient) and so on, and the factors associated with the 'flow' between these actions. This will involve observational visits, interviews with a range of clinical staff (e.g. consultants, registrars, pharmacists, nurses etc) and documentary analysis, which will be used to identify the actions required from all staff to (a) introduce SDD to the ICU and (b) maintain, regulate and optimise the delivery of SDD over time. These case studies will inform the practical issues around implementation of SDD in ICUs either in the context of a trial intervention or to inform implementation strategies. Two SDD units will be purposively sampled from an existing database from a national survey of SDD in the UK [15]. One Unit that has recently implemented SDD and one that has used SDD over a longer period will be identified. When identified, SDD Units will be contacted and asked to take part in the study. From multiple visits to each site, structured observations of the administration of SDD in the ICU will be followed by semi-structured interviews with nursing staff, clinical leads and other decision makers to elicit accounts of the processes of change, including decision making, documentation and resource issues. Retrospective accounts will be elicited, describing how the Unit decided to introduce SDD, factors that triggered change, barriers experienced (i.e., interruptions to the 'flow' of actions), and the strategies used to overcome them. The perceived consequences of the SDD policy for the unit (and the hospital more generally) will be documented.

The case studies will identify accounts of implementation of SDD in practice, in terms of the behaviours performed by the full range of individual clinicians; accounts of how SDD was first introduced into the Unit; specific content that may be used to populate the content of behaviour change techniques to be used in an implementation intervention and procedures to consider in order to deliver an implementation trial.

### Stage 2

#### A Delphi study to identify the range of stakeholders' beliefs, views and perceived barriers relating to the use of SDD (targeting RQs 3, 4,5 and 7)

This stage will be conducted in all three regions. This phase of the investigation will generate hypotheses about key beliefs about SDD and will inform the feasibility of any future randomised controlled trial. Using a Delphi technique comprising an initial exploratory 'round' so that the full range of views may be raised and then considered by all participants in later rounds, followed by three iterations (i.e. four rounds in all), we will sequentially build a picture of respondents' beliefs and views on SDD. We will further assess the likely factors influencing the acceptability of any future randomised controlled trial comparing SDD against a control group (not including SDD) in ICUs and/or a proposed randomised controlled trial comparing implementation strategies for SDD against a control group in ICUs. Rounds 1 to 3 will be conducted independently within each region, whilst round 4 will be multi-region.

There is a broad range of estimates of suitable sizes for a Delphi panel, but smaller sizes (such as 10 per stakeholder group) have been deemed appropriate where panel members have similar training [18]. Four key stakeholder clinician groups (ICU physicians, ICU pharmacists, infectious disease clinicians/medical microbiologists, ICU clinical managers) will be sampled using existing databases from within each region. We will aim for 10 from each stakeholder group. The total sample size will thus be approximately 40 in each of the three regions. Purposive diversity sampling will be used in all groups to identify as wide a range of initial views as possible, based on a range of variables: Academic-affiliated or not; years of experience (time since commencing as consultant/other professional grade); size of ICU (i.e. number of ICU beds); and current practice (routinely perform SDD or not). During the interview phase, diversity on these factors will be tracked using a diversity sampling table and additional participants will be invited to participate, if required, to maximise variation. Transcribed interviews will be content analysed based on the theoretical domains framework using methods previously employed by the research team in the context of critical care [19].

The Delphi study report will present (i) the perceived importance of each specific belief about the use of SDD in ICUs; (ii) the acceptability and feasibility of conducting an effectiveness trial; and (iii) the acceptability and feasibility of conducting an implementation trial. In round 4, the data from each regional setting will be presented to all international participants by email. The international comparisons in this round are important, to increase the generalisability of the results and of any future study result, since lack of generalisability may have been a major factor limiting the uptake and implementation of SDD in the past. This round will require that the investigations conducted in each country use a broadly parallel time frame, so that the feedback is presented to all participants with similar time intervals. The full Delphi results will allow us to consider how best to elaborate the key findings from the nationwide survey of the state of current practice, described below.

### Stage 3

#### Nationwide surveys of the state of current practice to identify the factors predicting acceptability of an effectiveness or implementation trial (targeting RQs 6 and 7)

This stage will be conducted in all three regions using large-scale postal questionnaire surveys using existing databases of intensivists and infectious disease clinicians/medical microbiologists in each region. This questionnaire design will be informed by the theoretical domains structure [17] but only the domains identified in the Delphi study as relevant to implementation of SDD will be included. Questionnaire items will be developed using standard theory-based methods with a 7-point response format [20]. The exact content of the questionnaire will be informed by the specific beliefs and views identified as important in the Delphi, thereby ensuring maximum relevance. The draft questionnaire (and cover letter) will be pilot tested using one-to-one interviews with 4 clinical collaborators to assess wording, acceptability and length. An appropriate course of action will then be agreed (i.e. accept, change, eliminate etc). Reminder letters (up to a maximum of two) will be posted to non-responders at two-week intervals after the first posting. Analysis will include simple descriptive and statistical prediction techniques [21-24]. These surveys will attempt to establish the current state of practice in ICUs with regard to SDD, the nature of evidence still required and perceived barriers to implementation of SDD. In addition, they will identify the willingness of intensive care clinicians to participate in any future effectiveness trial of SDD in intensive care practice and/or an implementation trial of strategies to increase uptake. The strongly predicting constructs and variables will be targeted if this programme progresses to an implementation trial, using systematic methods for intervention development [22].

### Stage 4

#### Semi-structured interviews with active clinical trialists in intensive care to assess the feasibility of a randomised controlled trial (targeting RQ 8)

Participants from all three regions as well as European investigators will take part in this stage. We will interview expert national and international clinical trialists in the intensive care field including trialists with experience in SDD to identify challenges and barriers to undertaking research in the field of SDD research. We will seek to recruit 10 participants from across the UK, Europe, Canada and Australia. Semi-structured one-to-one interviews using a topic guide developed from the previous phases as well as from expert experience will be used to study this area. Questions will address potential trial design issues (e.g. need for cluster randomisation), specification of the SDD intervention and of control group care; outcome measurement; recruitment; ethical considerations and other issues raised in the observational and Delphi studies. Data will be transcribed and analysed using content analysis [25]. A full description of the design and measurement issues to consider when planning a possible effectiveness trial will be produced.

### Outcome of project

This research will lead to an evidence-based decision about whether to proceed to a clinical trial to evaluate an SDD intervention or an implementation trial (i.e., development and evaluation of an intervention to increase uptake of SDD in ICUs). The applicant team/project steering group will make an assessment of whether a trial is necessary, justifiable, acceptable and feasible based on possible patterns of results from the national surveys, for example:

1. If intention to implement SDD is low or variable, and predicted by attitude scores and/or scores for specific beliefs about the consequences (benefits and harms) of implementing SDD, it will be judged appropriate to proceed to trial.

2. If intention to implement SDD is low or variable and predicted by scores relating to social influence (e.g. pressure from colleagues in other disciplines), this would suggest that an implementation intervention could be effective if delivered by an identified opinion leader, clinical lead or local 'champion' through team meetings. We would proceed to an implementation trial to evaluate such an intervention.

3. If intention to implement SDD is low or variable and predicted by beliefs relating to lack of capacity to implement SDD (e.g. resource issues), this would suggest that an implementation intervention could be effective if it focuses on barrier identification and generation of strategies to overcome barriers, known as 'coping planning' [26], we would proceed to an implementation trial to evaluate such an intervention.

4. If intention to implement SDD is low or variable and predicted (in the multi-level model) at the Unit level, rather than by individual clinicians' views of the evidence, we would design an intervention directed at ICUs rather than individual clinicians. This would be informed by the behavioural analysis of SDD implementation in ICUs where it is currently practised. We would proceed to an implementation trial to evaluate such an intervention.

5. By contrast, if intention to implement SDD is high (i.e., if there are ceiling effects and restricted variance), then the current low level of implementation will be attributable to an 'intention-behaviour gap', suggesting that external barriers prevent clinicians from translating their intentions into action. This pattern of results would suggest that an implementation trial to test strategies for facilitating uptake would be appropriate.

Findings about acceptability and feasibility of a clinical trial would also inform the decision about how best to proceed. Specifically:

1. If willingness (intention) to participate in a trial is low we will not proceed to a clinical trial.

2. If willingness (intention) to participate in a trial is high this will indicate that the trial is sufficiently acceptable to proceed. We will also take into account scores for perceived ease or difficulty of participating.

We will also use the consensus data from the Delphi study and the investigation of two ICUs where SDD has been implemented, to decide: (i) behavioural (practical, organisational and management) issues that would need to be addressed in order to mount a trial; (ii) ethical issues relating to informed acceptance of trial entry among eligible patients; (iii) trial design issues including measurement of outcome and process variables. If any one stakeholder group seems to be uniformly of the opinion that there are no design features that would make a trial acceptable, and/or if a third (or more) of the members of two (or more) groups deem any such trial unacceptable, the clinical trial should not be pursued further. Depending on the views expressed about the existing evidence base (i.e. if treatment is viewed as potentially beneficial) we will consider the place of an implementation trial to change clinical practice. If appropriate, the change techniques that would form the components of such a trial will be selected using methods previously reported by members of the research team [27,28]. The final decision on trial continuation will be made by the study steering committee.

## Discussion

The issue of hospital acquired infections in critically ill patients is a significant issue causing excess morbidity and mortality as well as an excess health care delivery costs. The intervention SDD has the promise to improve these outcomes and is not widely used. This rigorous multi-national study intends to identify the perceived risks, benefits and barriers to the use of SDD in critical care units. This research could lead to a variety of further interventions including a multi-centre effectiveness RCT or implementation study.

## Competing interests

The authors declare that they have no competing interests.

## Authors' contributions

BHC has participated fully in the design of this study and in the writing this paper and has seen and approved the final version of the paper. JF has participated fully in the design of this study and in the writing this paper and has seen and approved the final version of the paper.

MKC has participated fully in the design of this study and in the writing this paper and has seen and approved the final version of the paper. LM has participated fully in the design of this study and in the writing this paper and has seen and approved the final version of the paper. IS has participated fully in the design of this study and in the writing this paper and has seen and approved the final version of the paper. JG has participated fully in the design of this study and in the writing this paper and has seen and approved the final version of the paper.

## Authors' information

BHC is the chief of the Department of Critical Care Medicine, Sunnybrook Health Sciences Centre, Toronto and Professor of Anaesthesia at the University of Toronto. JF is an academic Health Psychologist at the University of Aberdeen, Aberdeen, UK. MKC is a clinical trialist and Director of the Health Services Research Unit at the University of Aberdeen, Aberdeen, UK. Lauralyn MacIntyre is a critical care specialist and associate scientist in clinical epidemiology at the Ottawa Health Research Institute, Ottawa, Canada. IS is a Staff Specialist in intensive care at the Nepean Hospital, Penrith, New South Wales, Australia. JMG is a Professor in the Department of Clinical Epidemiology at the Ottawa Health Research Institute, Ottawa, Canada.

The SuDDICU groups include-

SuDDICU UK study group - Jill Francis, International methods lead, Reader in Health Psychology, Health Services Research Unit, University of Aberdeen, Aberdeen, Scotland, UK; Geoff Bellingan, UK clinical lead, Director, Intensive Care Unit, University College Hospital, London, UK; Marion Campbell, Director, Health Services Research Unit, University of Aberdeen, Aberdeen, Scotland, UK; Brian Cuthbertson, Canadian and International lead, Chief, Department of Critical Care Medicine, Sunnybrook Health Sciences Centre, Toronto, Ontario, Canada; Martin Eccles, Professor of Clinical Effectiveness, University of Newcastle, Newcastle, UK; Marie Johnston, Professor of Health Psychology, Institute of Applied Health Sciences, University of Aberdeen, Aberdeen, Scotland, UK; Graeme MacLennan, Senior Statistician, Health Services Research Unit, University of Aberdeen, Aberdeen, Scotland, UK; Craig Ramsay, Director, Health Care Assessment Programme, Health Services Research Unit, University of Aberdeen, Aberdeen, Scotland, UK; Louise Rose, Assistant Professor of Nursing, University of Toronto, Toronto, Ontario, Canada; Kathy Rowan, Director and Professor, Intensive Care National Audit and Research Committee, London, UK; Rob Shulman, ICU Pharmacist, University College Hospital, London, UK.

SuDDICU Canadian study group - Brian Cuthbertson, Canadian and International lead, Chief, Department of Critical Care Medicine, Sunnybrook Health Sciences Centre, Toronto, Ontario, Canada; Jill Francis, International methods lead, Reader in Health Psychology, Health Services Research Unit, University of Aberdeen, Aberdeen, Scotland, UK; Karen Burns, Clinician Scientist and Attending Physician, St Michaels Hospital, Toronto, Ontario, Canada; Deborah Cook, Canada Research Chair in Critical Care, McMaster University, Hamilton, Ontario, Canada; Peter Dodeck, Center for Health Evaluation and Outcome Sciences, St. Paul's Hospital, Vancouver, British Columbia, Canada; Niall Ferguson, Assistant Professor and Attending Physician, University Health Network, Toronto, Ontario, Canada; Richard Hall, Professor of Anesthesiology and Pharmacology Associate Professor of Surgery Dalhousie University and The Queen Elizabeth II Health Sciences Centre, Halifax, Nova Scotia, Canada; Lynn Johnston, Professor of Medicine, Chief of Infectious Diseases, Dalhousie University, Halifax, Nova Scotia, Canada; Salmann Kanji, ICU pharmacist, Critical Care Unit, The Ottawa Hospital, Ottawa, Ontario, Canada; John Marshall, Professor of Surgery, University of Toronto and Attending Surgeon, St Michaels Hospital, Toronto, Ontario, Canada; Lauralyn McIntyre, Clinical Epidemiology, Ottawa Health Research Institute, Ottawa, Canada; John Muscedere, Associate Professor of Medicine, Queen's University, Intensivist Kingston General Hospital, Kingston, Ontario, Canada; Joe Pagliarello, University of Ottawa, Ottawa, Ontario, Canada; Louise Rose, Assistant Professor of Nursing, University of Toronto, Toronto, Ontario, Canada; Fiona Webster, Knowledge Translation Scientist, Sunnybrook Health Sciences Centre, Toronto, Ontario, Canada; Merrick Zwarenstein, Director Centre for Health Services Science, Sunnybrook Health Sciences Centre, Toronto, Ontario, Canada.

SuDDICU Australia and New Zealand Study Group - Ian Seppelt, Australia/New Zealand lead, Nepean Hospital, Penrith, New South Wales, Australia; Simon Finfer, Professor of Intensive Care and Staff Specialist, Royal North Shore Hospital and the George Institute, Sydney, Australia; Jeff Lipman, Professor and Head ICU, Royal Brisbane and Women's Hospital, Brisbane, Australia; John Myburgh, Professor, Intensive Care Unit, St George Hospital Sydney and the George Institute, Sydney, Australia; David Paterson, Professor Infectious Diseases and Microbiology, Royal Brisbane and Women's Hospital, Brisbane, Australia; Brian Cuthbertson, Canadian and International lead, Chief, Department of Critical Care Medicine, Sunnybrook Health Sciences Centre, Toronto, Ontario, Canada; Jill Francis, International methods lead, Reader in Health Psychology, Health Services Research Unit, University of Aberdeen, Aberdeen, Scotland, UK;
